# Ebola Virion Attachment and Entry into Human Macrophages Profoundly Effects Early Cellular Gene Expression

**DOI:** 10.1371/journal.pntd.0001359

**Published:** 2011-10-18

**Authors:** Victoria Wahl-Jensen, Sabine Kurz, Friedericke Feldmann, Lukas K. Buehler, Jason Kindrachuk, Victor DeFilippis, Jean da Silva Correia, Klaus Früh, Jens H. Kuhn, Dennis R. Burton, Heinz Feldmann

**Affiliations:** 1 Integrated Research Facility at Fort Detrick, Division of Clinical Research, National Institute of Allergy and Infectious Diseases, National Institutes of Health, Fort Detrick, Maryland, United States of America; 2 National Microbiology Laboratory, Canadian Science Centre for Human and Animal Health, Public Health Agency of Canada, Winnipeg, Manitoba, Canada; 3 Department of Medical Microbiology, University of Manitoba, Winnipeg, Canada; 4 The Scripps Research Institute, La Jolla, California, United States of America; 5 Integrated Research Facility at Rocky Mountain Laboratories, Division of Intramural Research, National Institute of Allergy and Infectious Diseases, National Institutes of Health, Hamilton, Montana, United States of America; 6 School of Math, Science and Engineering, Southwestern College, Chula Vista, California, United States of America; 7 Vaccine and Gene Therapy Institute, Oregon Health and Sciences University, Portland, Oregon, United States of America; 8 IAVI Neutralizing Antibody Center, The Scripps Research Institute, La Jolla, California, United States of America; 9 Ragon Institute of MGH, MIT and Harvard, Boston, Massachusetts, United States of America; University of Texas Medical Branch at Galveston, United States of America

## Abstract

Zaire ebolavirus (ZEBOV) infections are associated with high lethality in primates. ZEBOV primarily targets mononuclear phagocytes, which are activated upon infection and secrete mediators believed to trigger initial stages of pathogenesis. The characterization of the responses of target cells to ZEBOV infection may therefore not only further understanding of pathogenesis but also suggest possible points of therapeutic intervention. Gene expression profiles of primary human macrophages exposed to ZEBOV were determined using DNA microarrays and quantitative PCR to gain insight into the cellular response immediately after cell entry. Significant changes in mRNA concentrations encoding for 88 cellular proteins were observed. Most of these proteins have not yet been implicated in ZEBOV infection. Some, however, are inflammatory mediators known to be elevated during the acute phase of disease in the blood of ZEBOV-infected humans. Interestingly, the cellular response occurred within the first hour of Ebola virion exposure, i.e. prior to virus gene expression. This observation supports the hypothesis that virion binding or entry mediated by the spike glycoprotein (GP_1,2_) is the primary stimulus for an initial response. Indeed, ZEBOV virions, LPS, and virus-like particles consisting of only the ZEBOV matrix protein VP40 and GP_1,2_ (VLP_VP40-GP_) triggered comparable responses in macrophages, including pro-inflammatory and pro-apoptotic signals. In contrast, VLP_VP40_ (particles lacking GP_1,2_) caused an aberrant response. This suggests that GP_1,2_ binding to macrophages plays an important role in the immediate cellular response.

## Author Summary

Ebola virus causes a severe hemorrhagic fever syndrome in man with high case-fatality rates. Following infection, monocytes and macrophages are among the first cells targeted by the virus. These cells respond by increasing expression of inflammatory cytokines and chemokines that contribute towards pathogenesis. In order to more thoroughly characterize the host response to Ebola infection, primary human macrophages were infected with Zaire ebolavirus and samples harvested for transcriptional changes after 1 or 6 hours post infection. Whereas previous studies have analyzed a relatively small subset of host genes, this study examined the transcriptional profile of over 10,000 genes and employed rigorous pathway analyses to the datasets. Ebola virus was found to significantly regulate the expression of over 88 host genes. These changes occurred within the first hours of infection. Subsequent experiments demonstrated that virus replication was not necessary for activation. Indeed, noninfectious virus-like particles expressing the ebolavirus glycoprotein and matrix proteins were sufficient stimuli to induce activation.

## Introduction

Zaire ebolavirus (ZEBOV) is a member of the family *Filoviridae* within the order *Mononegavirales* [1]. It was discovered in 1976 in what is now the Democratic Republic of the Congo [2] as the etiological agent of a severe human viral hemorrhagic fever known as Ebola Hemorrhagic Fever (EHF). Infection with ZEBOV typically results in a rapidly fatal illness associated with high-level viremia, lack of an effective immune response, drastic lymphopenia, a severe coagulation disorder including disseminated intravascular coagulation and limited hemorrhages, widespread focal tissue necroses, systemic shock and multiorgan failure (reviewed in detail in [3]). While the pathogenesis of ZEBOV infection has been relatively well described in experimental animals [4], [5], only a few studies were reported that shed light on the molecular events following infection in humans. Unfortunately, these studies are partially contradictory. For instance, higher serum cytokine concentrations (IFN-α, IFN-γ, IL-2, IL-6, and TNF-α) were measured in seven fatally infected patients compared to two survivors in one study, suggesting that a hyperactive immune responses may contribute to fatal outcome [6]. Other studies, describing the responses of eight fatally infected patients and four survivors, did not reveal significant concentration differences of IFN-α, IL-2, and TNF-α. On the other hand, that study suggested that fatal infections are due to generalized immunosuppression, including decreased IFN-γ, IL-2, and IL-4 concentrations, lymphocyte apoptosis, and diminished IgG synthesis [7], [8], [9], [10]. The largest study to date included 42 fatally infected patients and 14 survivors. Hypersecretion of proinflammatory cytokines, chemokines and growth factors (IL-1β, IL-1RA, IL-6, IL-8, IL-15, IL-16, CXCL1 (GROα), CXCL10 (IP-10), eotaxin, M-CSF, MIP-1α, MIP-1β, MCP-1, MIF) and decreased concentrations of T lymphocyte-derived cytokines (IL-2, IL-3, IL-4, IL-5, IL-9, IL-13) concomitant to apoptotic loss of CD4 and CD8 T lymphocytes were typical for fatal cases [11]. Unfortunately, all these data reveal only the extent of homeostatic disarray in ZEBOV-infected individuals, but not its origin or genesis. It is therefore important to measure the responses of individual human cell types to infection, ideally in chronological order of their infection *in vivo*. Mononuclear phagocytes are very early, if not initial, targets of ZEBOV in humans and experimentally infected animals [12], [13], [14], [15]. *In vitro*, human and nonhuman primate macrophages are highly susceptible to ZEBOV infection with subsequent robust virus production [16], [17], suggesting they may be the major source of the high viremia observed during the critical stages of infection. Studies performed *in vitro* are also strongly indicative that macrophages play a major role in inducing cytokine/chemokine dysregulation. For instance, human monocytes and macrophages infected with ZEBOV react with increased expression of MCP-1, CXCL1, IL-1β, IL-6, IL-8, MIP-1α, RANTES and TNF-α [16], [18]. Previous studies revealed that similar increased levels of expression of some of these cytokines were triggered by incubation of human macrophages with Ebola virus-like particles (VLPs) consisting of the ZEBOV matrix protein VP40 and the spike glycoprotein (GP_1,2_) [19] or with UV-inactivated ZEBOV [16]. These findings indicated that virus replication might not be required for the activation of macrophages.

The aim of the current study was to determine the gene expression profiles of human macrophages exposed to infectious Ebola virions using DNA microarray technology (reviewed in [20], [21], [22], [23], [24]) and therefore elucidate virus-host interactions. Here, we determine the initial responses of human macrophages to Ebola virion exposure. In a first set of experiments, DNA microarray analysis was performed to determine gene expression profiles of human macrophages 1 h and 6 h after *in vitro* exposure to Ebola virions in comparison to mock-exposed cells. In parallel, macrophages were treated with LPS to assess the responsiveness of the cells and to compare the response to different stimuli as genes responding to both virions and LPS might highlight possible response pathways. In a second set of experiments, we distinguished between responses induced by virion binding/entry and responses that require virus gene expression, cellular signals occurring after virion entry by exposing macrophages to Ebola VLPs. We found that Ebola virions exposure, as well as exposure to VLPs can trigger most of the detected changes after 1 h of exposure, and thus independent of virus replication.

## Materials and Methods

### Cells and viruses

Primary human macrophages were obtained from two sources. For the first set of experiments, fresh elutriated primary human monocytes from three donors (D1, D2, D3) were purchased from Advanced Biotechnologies, Columbia, MD. For the second set of experiments, primary human monocytes from three donors (Poietics CD14^+^, untreated 2W-400 series) (D4, D5, D6) were purchased from Cambrex Bio Science Walkersville (Walkersville, MD). For differentiation of monocytes into macrophages, cells were cultivated in RPMI 1640 (Invitrogen, Carlsbad, CA) containing 20% heat-inactivated human AB serum (Sigma-Aldrich, St. Louis, MO), penicillin (100 U/ml), streptomycin (100 µg/ml), and L-glutamine (2 mM). Human embryonic kidney (HEK) 293T epithelial cells and grivet (*Chlorocebus aethiops*) kidney epithelial Vero E6 cells (ATCC, Rockville, MD) were maintained in DMEM (Invitrogen, Carlsbad, CA) containing 10% heat-inactivated fetal bovine serum (Invitrogen, Carlsbad, CA), penicillin (100 U/ml), streptomycin (100 µg/ml), and L-glutamine (2 mM). All cells were incubated at 37°C in a humidified 5% CO_2_ environment. The Mayinga strain of *Zaire ebolavirus* (ZEBOV), isolated in 1976 [2], was used for all infections, which were performed under biosafety level 4 conditions at the National Microbiology Laboratory of the Public Health Agency of Canada in Winnipeg, Manitoba. Prior to use, virus stocks were propagated in Vero E6 cells and clarified by centrifugation at 3,000 *g* for 10 min at 4°C. Supernatants were then layered on TNE buffer (20 mM Tris [pH 7.5], 0.1 M NaCl, 0.1 mM EDTA) containing 20% sucrose and spun at 28,000 rpm at 4°C for 2 h by using an SW28 rotor with a Beckman Optima L-70K ultracentrifuge. The virion pellet was resuspended in RPMI 1640 and titers were determined by plaque assay as previously described [25]. As a control, supernatant from mock-infected Vero E6 cells was purified and quantified by the same procedures.

### Generation of virus-like particles

Generation and purification of virus-like particles (VLPs) were performed as described elsewhere [19]. In brief, ZEBOV VLP_VP40-GP_ and VLP_VP40_ were generated by transient transfection of HEK 293T cells with plasmids encoding ZEBOV VP40 and/or ZEBOV GP_1,2_ [19] and quantitated by electron-microscopic particle counting [26] and a DC protein assay (BioRad, Mississauga, Ontario).

### Negative-stain electron microscopy

Electron-microscopic evaluation of VLPs was performed on a Phillips CM100 microscope with low dose software and Compustage attachments. Negative staining was performed on formvar carbon-coated copper grids (Electron Microscopy Sciences, Hatfield, PA). Purified VLP solution (13 µl) was exposed to a freshly glow-discharged grid for 2 min, and the grid then transferred to a drop of 1% sodium silicotungstate (pH 7.5) for 1 min. The liquid was carefully removed by applying Whatman® paper at the edge of the grid. The grid was air dried for at least 1 h before electron-microscopic examination. Images were recorded at machine magnifications of 56,000× and 72,000× on Direct Positive Film 5302 (Kodak, Rochester, NY).

### Endotoxin test

Prior to use, virion stocks, mock stocks, VLPs, and media were analyzed for endotoxin presence using the *Limulus* amebocyte lysate test (BioWhittaker, Walkersville, MD).

### Exposure of macrophages

Six days after seeding, growth medium of human macrophages was replaced with fresh RPMI 1640 containing 2% human AB serum, penicillin (100 U/ml), streptomycin (100 µg/ml), and L-glutamine (2 mM). Cells were then incubated for one day at 37°C in a humidified, 5% CO_2_ environment. Cells from donors D1, D2, and D3 were infected with either mock virus preparation or ZEBOV at a multiplicity of infection (MOI) of 10. Cells of donors D4, D5, and D6 were infected with ZEBOV at an MOI of 100, mock virus preparation, ∼100 particles/cell VLP_VP40-GP_, ∼100 particles per cell VLP_VP40_, mock VLP (plasmid only), ∼100 latex particles/cell, or 10 ng/ml of lipopolysaccharide (LPS). Cell supernatants were removed from the cells 1 h or 6 h post infection, and RNA from cells was purified using the RNeasy Mini Kit (QIAGEN, Valencia, CA) according to the manufacturer's instructions. The analysis of purified RNA from 10 MOI was analyzed by DNA microarrays as outlined below. The RNA obtained after infection with 100 MOI was subjected to quantitative real-time RT-PCR analysis only.

### Microarrays

Genechip technology from Affymetrix (Santa Clara, CA) was used to study the transcriptional activity of human genes using the human GeneChip® array HG-U95Av2 with a total of 12,626 probes representing approximately 10,000 full-length genes (Affymetrix Technical Note to Human Genome U133 Genechip set). Normalized signal values were generated from image raw data and used to calculate *p* values indicating significance levels for signal strength (absent or present call) and log_2_ ratio values in comparison files (change, increase, decrease calls) using the Affymetrix Microarray Suite 5.0 (MAS 5.0). Analysis was performed on these normalized data with a reduced set of genes after removing all genes that were absent on all arrays. The remaining data included 8,861 probe sets. Additional data reduction was achieved by excluding all genes that were identified as ‘no change’ (NC) in all six experiments when comparing Ebola virion-exposed with mock-exposed cells, respectively. The remaining dataset included 2,025 upregulated or downregulated genes. An analysis of variance (single factor ANOVA) and determination of the correlation coefficient (R^2^) of changes in gene expression levels between ZEBOV-treated and VLP-treated cells was performed using Microsoft Office Excel. Array data were displayed on MA plots (Figure S1), i.e. on a scatter plot showing the correlation between the average log_2_ intensity versus log_2_ ratio for a ZEBOV-treated versus mock-treated pair of cells. Log intensity was calculated as ½ (log(virion)+log(mock)). Genes unchanged between test and control have a log_2_ value of zero; downregulated genes have negative values; and upregulated genes have positive values. Hierarchical clustering was performed using Stanford's GeneCluster and displayed with the TreeView program [27]. Clustering selection used average linkage clustering with the correlation uncentered.

### Pathway analysis of differentially expressed genes

InnateDb (www.innatedb.com) is a publically available resource which, based on levels of either differential gene expression, predicts biological pathways based on experiment fold change datasets [28]. Pathways are assigned a probability value (p) based on the number of genes present for a particular pathway as well as the degree to which they are differentially expressed or modified relative to a control condition. For our investigation input data was limited to the subset of 2,025 genes identified above. Additionally, functional networks were created using Ingenuity Pathway Analysis (IPA) software (Ingenuity Systems, Redwood City, CA). Those genes with known gene symbols and their corresponding expression values were uploaded and mapped to their corresponding gene objects in the IPA Knowledge Base. Networks of these genes were algorithmically generated based on their connectivity and assigned a score. Genes are represented as nodes, and the biological relationship between two nodes is represented as an edge (line). The intensity of the node color indicated the degree of up- or down-regulation. Genes in uncolored notes were not identified as differentially expressed in our experiment and were integrated into the computationally generated networks on the basis of the evidence stored in the IPA knowledge memory indicating a relevance to this network.

### Relative quantification of transcript levels by real-time PCR

Reverse transcription and subsequent quantitative real-time polymerase chain reaction (qPCR) were performed as described previously [19]. Strand-specific qPCR was performed using strand-specific primers for the reverse transcriptase reaction. As controls for non-specific or self-priming events, control reverse transcriptase reactions lacking primer were performed in parallel. Relative amounts of different strands were determined by normalizing against the house-keeping gene GAPDH and by subtracting the amounts of PCR product resulting from self-priming from strand-specific products. Relative quantification was performed using the comparative CT method (Applied Biosystems User Bulletin #2, Dec. 11, 1997).

## Results

### DNA microarray data analysis

DNA microarray technology was used to determine the initial response of human macrophages exposed to Ebola virions. Total RNA isolated from primary human macrophages of three donors (D1, D2, D3) at 1 h and 6 h after *in vitro* exposure to purified Ebola virions was compared to RNA from mock-exposed cells derived from the same donors. The time points were chosen as virus gene expression does not occur within one hour of cell-virion contact, whereas it will have commenced five hours later while ZEBOV replication is still absent (see below). A total of 12 HG-U95Av2 GeneChip microarrays were analyzed to determine differences in cellular gene expression levels between Ebola virion-exposed and mock-exposed macrophages from donors D1–D3 and also compared to the responses of LPS-treated cells. Genes detected as absent (*p*≤0.05) on all 12 arrays were removed from a total of 12,606 probe sets prior to ratio analysis resulting in a reduced number of 8,861 probe sets. The log_2_ ratio was plotted as a function of the average log_2_ intensity of Ebola virion-exposed versus mock-exposed samples to test for hybridization quality and for the extent of variation among biological replicates (cells from the three donors). The MA plots for both the 1 h and 6 h time points demonstrate a low variability among donors and good overall reproducibility with the mean log_2_ being zero over the entire signal intensity range (Figure S1). The low overall variability among donors was corroborated quantitatively by an analysis of variance of the signal strength. A single factor analysis of variance (ANOVA) indicated no significant difference among the 12 arrays (*p* = 0.83), confirming that the expression of the majority of cellular genes was not affected by exposure to Ebola virions. Genes responsive to Ebola virion exposure were identified by data reduction, i.e. by exclusion of all genes that were identified as ‘no change’ (NC, *p*≤0.05) for all conditions tested. The resulting 2,025 probe sets (genes) were characterized by at least one significant change in one donor at either 1 h or 6 h post infection. ANOVA indicated that differences in expression patterns among these probe sets were statistically significant (*p* = 3×10^−6^).

**Figure 1:**
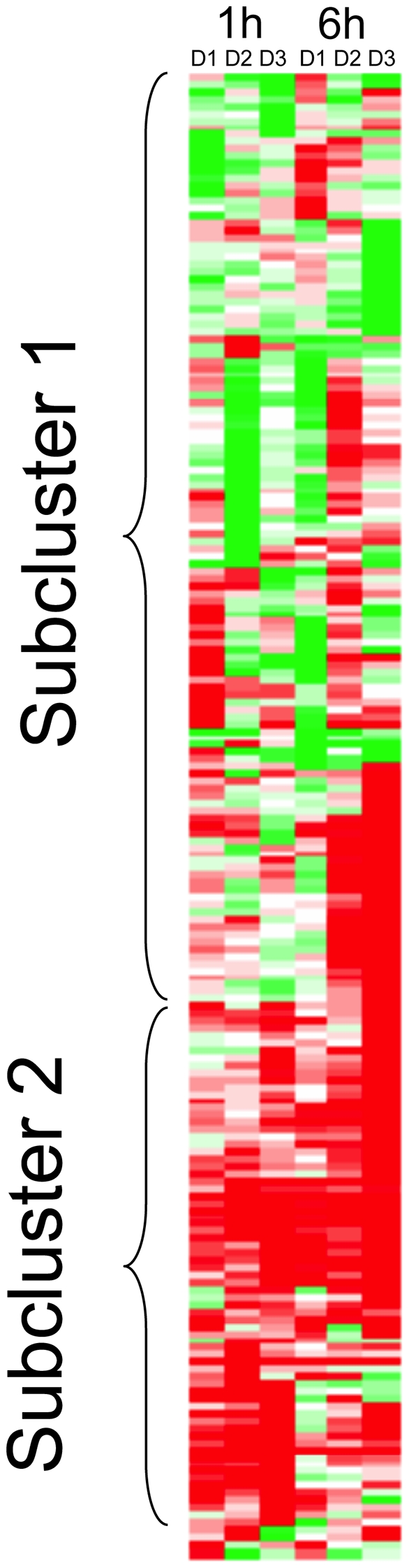
Color-coded hierarchical cluster analysis. Threshold Criteria: changes of gene expression levels in primary macrophages of at least 2-fold (“threshold”) occurring in at least two of the three donors in at least one of the two time points upon Ebola virion exposure compared to mock exposure. Gene subcluster 1 is characterized by expression variability across donors and time points. Subcluster 2 is characterized by consistent responses among all 3 donors in at least one time point.

**Figure 2:**
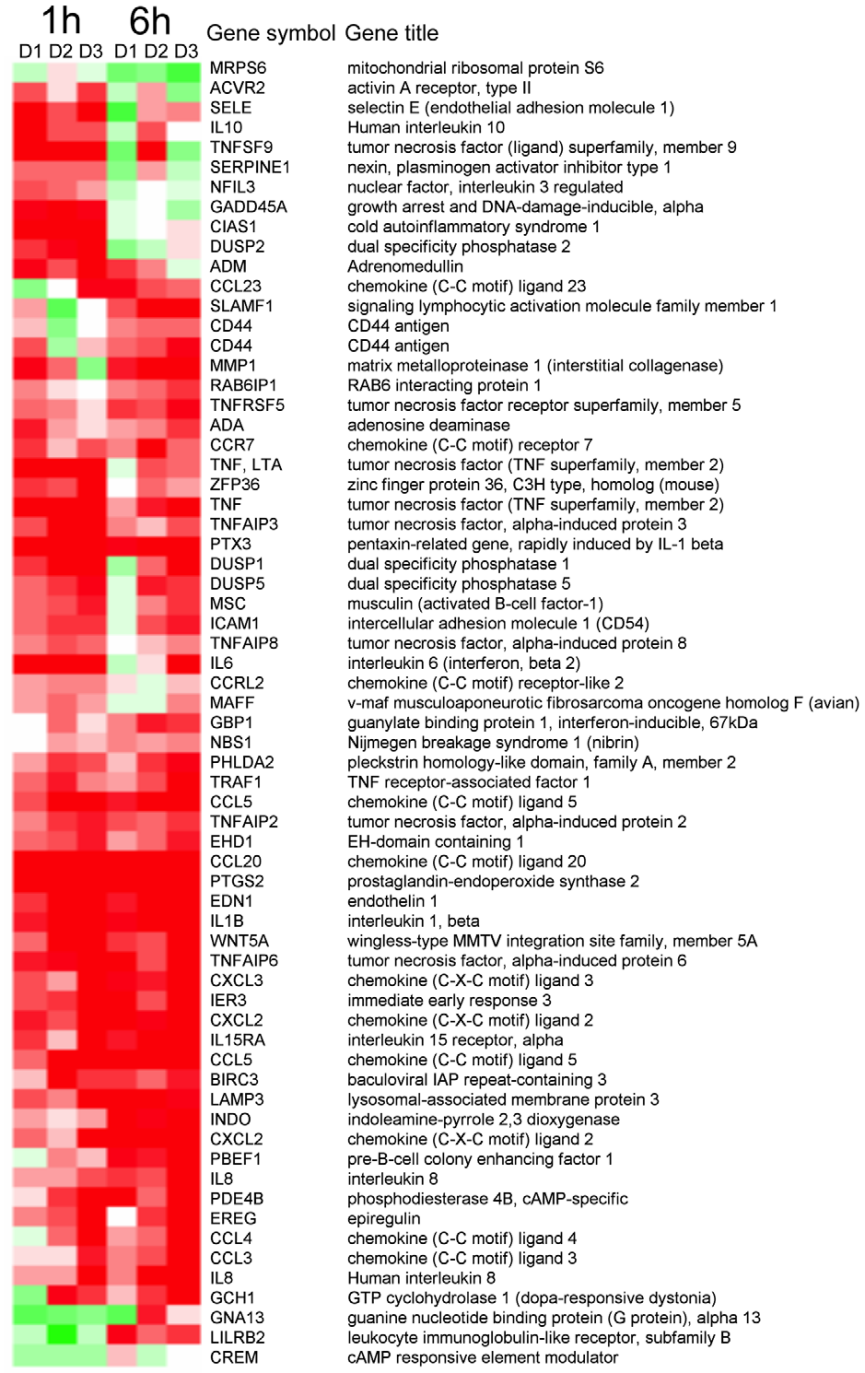
Cluster analysis of genes selected by Trend Criteria. Changes of gene expression levels occurred in primary macrophages from all three donors (“trend”) in at least one time point in the same direction with *p*-values<0.01. Red and green colors represent upregulation and downregulation, respectively. Intensity of colors reflects intensity of changes in gene expression.

The 2,025 genes identified were then screened for patterns of cellular expression changes that could be biologically relevant. Two types of selection criteria were designed to determine whether expression of a cellular gene was significantly affected by Ebola virion exposure, threshold-based criteria and trend-based criteria. First, thresholds for fold-change, *p*-values, and signal strength were established. Specifically, a minimum of a 2-fold-difference in cellular gene expression levels in infected macrophages from at least two of the three donors in at least one of the two time points and *p*-values<0.05 were defined as pertinent (Figure 1). Second, it was acknowledged that in the case of most genes the extent of changes in gene expression required for a biological impact are unknown. For instance, some genes might respond to virus infection with only minor changes in expression levels that may still be biologically relevant. Vice versa, rather dramatic changes may prove to be biologically irrelevant. Consequently, trend selection criteria were established to screen for a common tendency or direction of changes in cellular gene expression levels. Only changes with an associated *p*-value of ≤0.01 were considered, and no unique cutoff value for fold-change was specified. Accordingly, changes of less than 2-fold were accepted if the direction of change in macrophages from all donors was the same (all increased or all decreased). The two resulting gene sets were analyzed by hierarchical clustering (Stanford Cluster software) and the resulting clusters were visualized using TreeView (Figures 1 & 2).

Application of the threshold-based selection criteria identified 205 cellular genes whose expression changed upon Ebola virion exposure (Figure 1). Clustering of these 205 genes revealed two subclusters. Gene subcluster 1 was characterized by extensive expression variability across donors and time points. Inspection of transcript levels (signal strength) revealed very low signals, which may be a reason of the apparent variability among biological replicates. In contrast, many genes in subcluster 2 show consistent responses among all three donors in at least one time point. However, there are some genes that show similar responses in cluster 1. Since the overall variability in subcluster 1 could be due to low signal strength the dataset of 205 genes was subjected to the additional requirement that of the two compared signal levels the higher one (i.e. mock-signal for upregulated genes, Ebola-signal for downregulated genes) has a minimum signal value of 100 (signal threshold). Fifty-one genes remained when this added threshold criterion was applied. Most of them were located in subcluster 2 and are listed in Table S1 (threshold selection). The majority of the 51 genes were upregulated at 1 h post infection, at 6 h, or at both time points.

The second set of criteria, designed for selecting a trend of responses rather than assuming a threshold for fold-change, yielded a total of 66 probe sets (63 genes) (see Figure 2 and Table S1, trend selection). Approximately half of them were characterized by a fold-change of ≥2 and were also identified after the first set of selection criteria was applied. A total of 88 genes were identified after either criterion was applied. Among those 88 genes from threshold and trend analysis (Table S1), 26 fulfilled both selection criteria. Twenty-one of these genes were characterized by altered expression levels following exposure to Ebola virions in the same direction as after treatment with LPS. Interestingly, the expression levels of cellular genes identified by applying the trend analysis (Table S1 & Figure 2), all changed at the 1 h time point. Importantly, this set includes genes whose elevated expression was previously associated with human Ebola virus disease, such as the genes encoding CXCL1, IL-1β, IL-6, IL-8, and TNF-α [6], [11], as well as genes previously unknown to play a role in Ebola virus disease, such as those encoding CCL-20, COX-2, IL-15 receptor α, phosphodiesterase 4B, and t-Pa. Expression levels of almost all identified genes were upregulated, with the exception of genes encoding GNA13, CREM, and LILRB2 at the 1 h time point and MRPS6, GADD45, and DUSP2 at the 6 h time point.

### Pathway over-representation analysis of gene expression data

**Figure 3:**
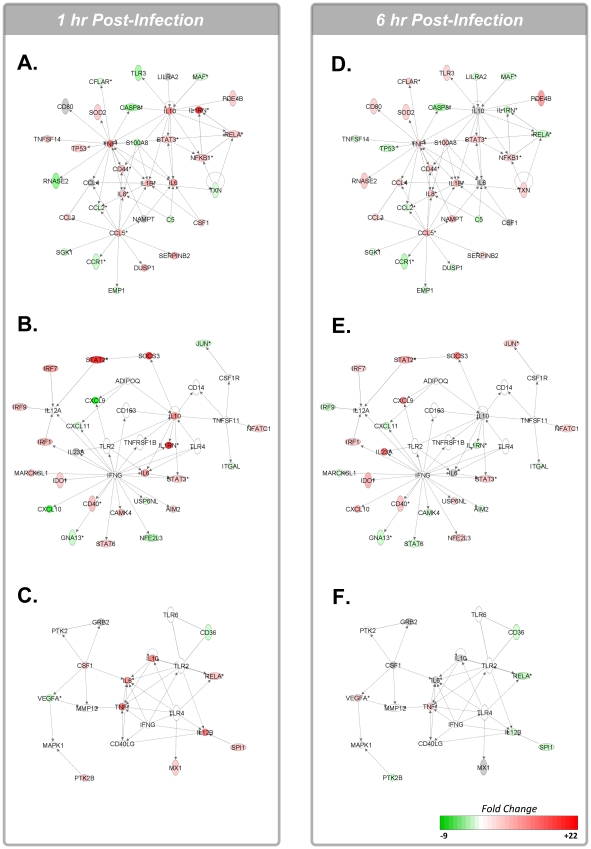
Functional networks associated with differentially expressed host gene expression 1 h and 6 h post-infection. The intensity of the node color indicates the degree of up (red)- or down (green)-regulation. Genes in uncolored nodes were not identified as differentially expressed in our experiment and were integrated into the computationally generated networks on the basis of the evidence stored in the IPA knowledge memory indicating a relevance to this network. A. Network 1 at 1 h post-infection - Cell-To-Cell Signaling and Interaction, Hematological System Development and Function, Immune Cell Trafficking; B. Network 2 at 1 h post-infection – Inflammatory Response, Cellular Movement, Hematological System Development and Function; C. Network 3 at 1 h post-infection – Cellular Movement, Hematological System Development and Function, Immune Cell Trafficking. D. Network 1 at 6 h post-infection - Cell-To-Cell Signaling and Interaction, Hematological System Development and Function, Immune Cell Trafficking; E. Network 2 at 6 h post-infection – Inflammatory Response, Cellular Movement, Hematological System Development and Function; F. Network 3 at 6 h post-infection – Cellular Movement, Hematological System Development and Function, Immune Cell Trafficking.

Recently, the integration of bioinformatics to complex biological data sets has provided a network-based approach for delineating the host response. As cellular responses are mediated through the selective activation or repression of signaling pathways we sought to integrate our gene expression data into functional signaling networks. Functional networks were created from our biological data sets using Ingenuity Pathway Analysis (IPA) (Figure 3). Genes belonging to these functional networks were related to cell-to-cell signaling and interactions, hematological system development and function, immune cell trafficking, inflammatory response and cell movement. To expand on the biological significance of this analysis, and identify individual signaling pathways modulated following infection, we performed pathway over-representation analysis (ORA) with the online software tool InnateDB (www.innatedb.com) [28] and analyzed the 1 h and 6 h Ebola-infected vs. mock-infected comparative data sets. Integrated data was limited to the 2,025 genes identified above and fold-changes >1.5 and associated p-values>0.01 were chosen as parameters for pathway ORA. The resultant differentially regulated pathways with p-values<0.1 are presented in Tables 1 and 2.

**Table 1:** Differential host signaling pathway modulation in Ebola-infected cells 1 h post-infection.

Pathway Name	Source Name	Genes in Pathway	Genes With Increased Expression	Up-Regulated PathwayP Value	Genes With Decreased Expression	Down-Regulated PathwayP Value
Heterotrimeric GPCR signaling pathway (through G alpha s ACs PKA BRaf and ERK cascade)(canonical) (GPCR signaling (G alpha s, PKA and ERK))	INOH	43	16	0.00088	4	0.93421
Heterotrimeric GPCR signaling pathway (through G alpha i and pertussis toxin) (GPCR signaling (pertussis toxin))	INOH	37	14	0.00156	3	0.95254
Heterotrimeric GPCR signaling pathway (through G alpha s ACs Epac BRaf and ERKcascade) (GPCR signaling (G alpha s, Epac and ERK))	INOH	37	14	0.00156	3	0.95254
Heterotrimeric GTP-binding protein coupled receptor signaling pathway (through G alpha i, adenylate cyclase and cAMP) (GPCR signaling (G alpha i))	INOH	37	14	0.00156	3	0.95254
Heterotrimeric GTP-binding protein coupled receptor signaling pathway (through G alpha s, cholera toxin, adenylate cyclase and cAMP) (GPCR signaling (cholera toxin))	INOH	40	14	0.00370	3	0.96827
Heterotrimeric GPCR signaling pathway (through G alpha q, PLC beta and ERK cascade) (GPCR signaling (G alpha q))	INOH	42	14	0.00613	2	0.99481
Dilated cardiomyopathy	KEGG	15	7	0.00674	2	0.72108
Jak-STAT signaling pathway	KEGG	47	15	0.00725	3	0.98807
Arrhythmogenic right ventricular cardiomyopathy (ARVC)	KEGG	12	6	0.00814	1	0.87865
IL27-mediated signaling events	PID NCI	13	6	0.01302	0	1
ATF-2 transcription factor network	PID NCI	17	7	0.01507	1	0.95001
African trypanosomiasis	KEGG	17	7	0.01507	0	1
Graft-versus-host disease	KEGG	7	4	0.01818	1	0.70683
Calcium signaling in the CD4+ TCR pathway	PID NCI	11	5	0.02529	0	1
Cytokine-cytokine receptor interaction	KEGG	73	19	0.02729	8	0.92362
Amine ligand-binding receptors	REACTOME	2	2	0.02846	0	1
Dissolution of Fibrin Clot	REACTOME	2	2	0.02846	0	1
Dual incision reaction in TC-NER	REACTOME	2	2	0.02846	0	1
Fibrinolysis pathway	PID BIOCARTA	2	2	0.02846	0	1
Formation of transcription-coupled NER (TC-NER) repair complex	REACTOME	2	2	0.02846	0	1
IL-2 signaling pathway (JAK1 JAK3 STAT5) (IL-2 signaling (JAK1 JAK3 STAT5))	INOH	2	2	0.02846	0	1
Multi-drug resistance factors	PID BIOCARTA	2	2	0.02846	0	1
Allograft rejection	KEGG	8	4	0.03163	1	0.75415
IL12-mediated signaling events	PID NCI	24	8	0.03646	2	0.91823
Class B/2 (Secretin family receptors)	REACTOME	5	3	0.03654	0	1
IL23-mediated signaling events	PID NCI	20	7	0.03829	1	0.97070
Malaria	KEGG	20	7	0.03829	1	0.97070
Negative feedback regulation of JAK STAT pathway by (cytokine receptor degradation signaling) (JAK-STAT pathway and regulation pathway Diagram)	INOH	21	7	0.04948	1	0.97549
Type I diabetes mellitus	KEGG	9	4	0.04956	1	0.79387
Coregulation of Androgen receptor activity	PID NCI	10	1	0.84457	5	0.01268
S1P4 pathway	PID NCI	4	0	1	3	0.01430
Amine compound SLC transporters	REACTOME	2	0	1	2	0.02556
Na+/Cl− dependent neurotransmitter transporters	REACTOME	2	0	1	2	0.02556
Overview of telomerase rna component gene hterc transcriptional regulation	PID BIOCARTA	2	0	1	2	0.02556
Regulation of CDC42 activity	PID NCI	2	0	1	2	0.02556
Metabolism of non-coding RNA	REACTOME	8	0	1	4	0.02632
SnRNP Assembly	REACTOME	8	0	1	4	0.02632
N-Glycan biosynthesis	KEGG	5	0	1	3	0.03156
Negative regulation of (G alpha i GDP-GTP exchange signaling) (GPCR signaling (G alpha i))	INOH	5	0	1	3	0.03156
Negative regulation of (G alpha i GDP-GTP exchange signaling) (GPCR signaling (pertussis toxin))	INOH	5	0	1	3	0.03156
RNA transport	KEGG	21	1	0.98041	7	0.03816
CXCR3-mediated signaling events	PID NCI	9	1	0.81260	4	0.04155
Metabolism of RNA	REACTOME	13	1	0.91143	5	0.04308

Differential host signaling pathway modulation identified by pathway ORA through the direct comparison of mock-infected and EBOV-infected macrophages 1 hr post-infection. The online software InnateDB was utilized for pathway over-representation analysis. Based on levels of differential expression InnateDB is able to predict pathways that are consistent with the experimental data. Pathways are assigned a probability value (p) based on the number of proteins present for a particular pathway. It also provides the number of uploaded pathways associated with a particular pathway as well as the subset of individual genes that are differentially expressed.

**Table 2:** Differential host signaling pathway modulation in Ebola-infected cells 6 h post-infection.

Pathway Name	Source Name	Genes in Path-way	Genes With Increased Expression	Up-Regulated PathwayP Value	Genes With Decreased Expression	Down-Regulated PathwayP Value
Syndecan-1-mediated signaling events	PID NCI	5	5	0.00023	0	1
Cytokine-cytokine receptor interaction	KEGG	73	25	0.00095	7	0.99528
Signaling by GPCR	REACTOME	56	20	0.00178	11	0.56426
Chemokine signaling pathway	KEGG	55	19	0.00362	5	0.99167
Nicotinate and Nicotinamide metabolism	INOH	5	4	0.00536	0	1
Peptide ligand-binding receptors	REACTOME	20	9	0.00644	4	0.57903
Prostaglandin and Leukotriene metabolism	INOH	3	3	0.00673	0	1
Chemokine receptors bind chemokines	REACTOME	12	6	0.01446	2	0.71951
Nicotinate and nicotinamide metabolism	KEGG	4	3	0.02315	0	1
G alpha (q) signalling events	REACTOME	21	8	0.03083	2	0.94103
Cardiac muscle contraction	KEGG	2	2	0.03581	0	1
Lysosphingolipid and LPA receptors	REACTOME	2	2	0.03581	0	1
Regulation of CDC42 activity	PID NCI	2	2	0.03581	0	1
Regulation of beta-cell development	REACTOME	2	2	0.03581	0	1
CD4 T cell receptor signaling (through Vav, Rac and JNK cascade (CD4 T cell receptor signaling (JNK cascade))	INOH	11	5	0.04013	2	0.67174
Antigen processing and presentation	KEGG	8	4	0.04658	2	0.48975
Arachidonic acid metabolism	KEGG	5	3	0.04984	1	0.66788
Steroid hormone biosynthesis	KEGG	5	3	0.04984	1	0.66788
RNA Polymerase I Transcription	REACTOME	5	0	1	4	0.00626
ATP+ADP = ADP+ATP (Purine nucleotides and Nucleosides metabolism)	INOH	3	0	1	3	0.00760
ATP+CDP = ADP+CTP (Pyrimidine Nucleotides and Nucleosides metabolism)	INOH	3	0	1	3	0.00760
ATP+GDP = ADP+GTP (Folate metabolism)	INOH	3	0	1	3	0.00760
						
ATP+GDP = ADP+GTP (Purine nucleotides and Nucleosides metabolism)	INOH	3	0	1	3	0.00760
ATP+IDP = ADP+ITP (Purine nucleotides and Nucleosides metabolism)	INOH	3	0	1	3	0.00760
ATP+UDP = ADP+UTP (Pyrimidine Nucleotides and Nucleosides metabolism)	INOH	3	0	1	3	0.00760
ATP+dADP = ADP+dATP (Purine nucleotides and Nucleosides metabolism)	INOH	3	0	1	3	0.00760
ATP+dCDP = ADP+dCTP (Pyrimidine Nucleotides and Nucleosides metabolism)	INOH	3	0	1	3	0.00760
ATP+dGDP = ADP+dGTP (Purine nucleotides and Nucleosides metabolism)	INOH	3	0	1	3	0.00760
ATP+dIDP = ADP+dITP (Purine nucleotides and Nucleosides metabolism)	INOH	3	0	1	3	0.00760
ATP+dTDP = ADP+dTTP (Pyrimidine Nucleotides and Nucleosides metabolism)	INOH	3	0	1	3	0.00760
ATP+dUDP = ADP+dUTP (Pyrimidine Nucleotides and Nucleosides metabolism)	INOH	3	0	1	3	0.00760
Post-Elongation Processing of Intronless pre-mRNA	REACTOME	3	0	1	3	0.00760
Processing of Capped Intronless Pre-mRNA	REACTOME	3	0	1	3	0.00760
Processing of Intronless Pre-mRNAs	REACTOME	3	0	1	3	0.00760
Transcription	REACTOME	20	2	0.91737	9	0.00852
RNA Polymerase I, RNA Polymerase III, and Mitochondrial Transcription	REACTOME	9	0	1	5	0.01808
Cleavage of Growing Transcript in the Termination Region	REACTOME	4	0	1	3	0.02597
MRNA 3′-end processing	REACTOME	4	0	1	3	0.02597
Post-Elongation Processing of Intron-Containing pre-mRNA	REACTOME	4	0	1	3	0.02597
Post-Elongation Processing of the Transcript	REACTOME	4	0	1	3	0.02597
RNA Polymerase I Promoter Clearance	REACTOME	4	0	1	3	0.02597
RNA Polymerase I Transcription Initiation	REACTOME	4	0	1	3	0.02597
RNA Polymerase I Transcription Termination	REACTOME	4	0	1	3	0.02597
RNA Polymerase II Transcription Termination	REACTOME	4	0	1	3	0.02597
Stabilization and accumulation of cytoplasmic beta-catenin (Canonical) (Canonical Wnt signaling pathway Diagram)	INOH	4	1	0.56935	3	0.02597
Stabilization and accumulation of cytoplasmic beta-catenin (Canonical) (Mammalian Wnt signaling pathway Diagram)	INOH	4	1	0.56935	3	0.02597
Stabilization and accumulation of cytoplasmic beta-catenin (Mammal) (Mammalian Wnt signaling pathway Diagram)	INOH	4	1	0.56935	3	0.02597
L1CAM interactions	REACTOME	7	1	0.77162	4	0.03141
Rb tumor suppressor/checkpoint signaling in response to dna damage	PID BIOCARTA	7	0	1	4	0.03141
Neuroactive ligand-receptor interaction	KEGG	28	6	0.44392	10	0.03403
Activated TAK1 mediates p38 MAPK activation	REACTOME	2	0	1	2	0.03883
Amine ligand-binding receptors	REACTOME	2	0	1	2	0.03883
Glutathione conjugation	REACTOME	2	0	1	2	0.03883
Leukotriene synthesis	REACTOME	2	0	1	2	0.03883
Multi-drug resistance factors	PID BIOCARTA	2	0	1	2	0.03883
Nef-mediates down modulation of cell surface receptors by recruiting them to clathrin adapters	REACTOME	2	0	1	2	0.03883
Netrin mediated repulsion signals	REACTOME	2	0	1	2	0.03883
Polyadenylation of mrna	PID BIOCARTA	2	0	1	2	0.03883
Recycling of bile acids and salts	REACTOME	2	0	1	2	0.03883
Synthesis of bile acids and bile salts via 7alpha-hydroxycholesterol	REACTOME	2	0	1	2	0.03883

Differential host signaling pathway modulation identified by pathway ORA through the direct comparison of mock-infected and EBOV-infected macrophages 6 hr post-infection. The online software InnateDB was utilized for pathway over-representation analysis as in Table 1.

Pathway ORA of the data set for the 1 h Ebola-infected vs. mock-infected treatments identified a number of pathways directly related to activation of the G-protein coupled receptor pathway (Table 1). As chemokines and inflammatory mediators activate GPCR signaling this correlates with previous investigations demonstrating increased secretion of cytokines and chemokines following Ebola virus insult [16], [18]. It was also demonstrated that many of the genes identified as central nodes in the IPA functional networks (IL-6, IL-10, IRF-7, etc.) also occupied central positions in the signaling pathways identified with InnateDB (heterotrimeric GPCR signaling pathways). Indeed, the upregulation of pathways related to interleukin (IL)-2, IL-12, IL-23 and IL-27 signaling pathways were also identified as being differentially upregulated in Ebola-infected cells as compared to the mock-infected treatment (Table S1). Further, GPCR-related signaling pathways (cytokine-cytokine receptor interaction pathway) and the Jak-Stat signaling pathway were also upregulated during the immediate response to infection. Interestingly, pathways related to fibrinolysis (dissolution of fibrin clot pathway; fibrinolysis pathway) were also upregulated by Ebola infection as compared to the mock-infected control (Table 1). Previously, dysregulation of the fibrinolytic system was recognized in Ebola-infected macaques [13]. A limited number of downregulated pathways were identified in our analysis as being significantly downregulated at 1 h post-infection and were largely related to metabolic processes as well as inhibition of the negative regulation of GPCR signaling (Table 1).

In contrast, differentially upregulated signaling responses at the 6 h time point were largely related to cell adhesion (syndecan-1-mediated signaling events), metabolism (prostaglandin and leukotriene metabolism; arachidonic acid metabolism; steroid hormone metabolism) and cytokine/chemokine signaling (cytokine-cytokine receptor interaction; chemokine signaling pathway; chemokine receptors bind chemokines) (Table 2). Interestingly, there was a large increase in the number of downregulated pathways at the 6 h time point as compared to the 1 h time point. Multiple pathways belonging to transcription and nucleotide/nucleoside metabolism were identified as being significantly downregulated in Ebola-infected cells as compared to the mock-infected controls 6 h post-infection (Table 2). Comparison of the differentially regulated pathway ORA data sets between the 6 h Ebola-infected vs. mock-infected and LPS-stimulated vs. mock-stimulated treatments demonstrated minimal overlap of upregulated or downregulated pathways (data not shown). Whereas LPS stimulation resulted in the upregulation of pathways largely related to TNF-α, Toll-like receptor (TLR) or apoptosis signaling pathways these were not identified in the Ebola-infected samples. Thus, this likely indicates that the pro-inflammatory response to Ebola may be largely repressed by a yet unidentified mechanism at early time points following infection.

### Real-time RT-PCR quantification of changes in cellular gene expression levels

**Figure 4:**
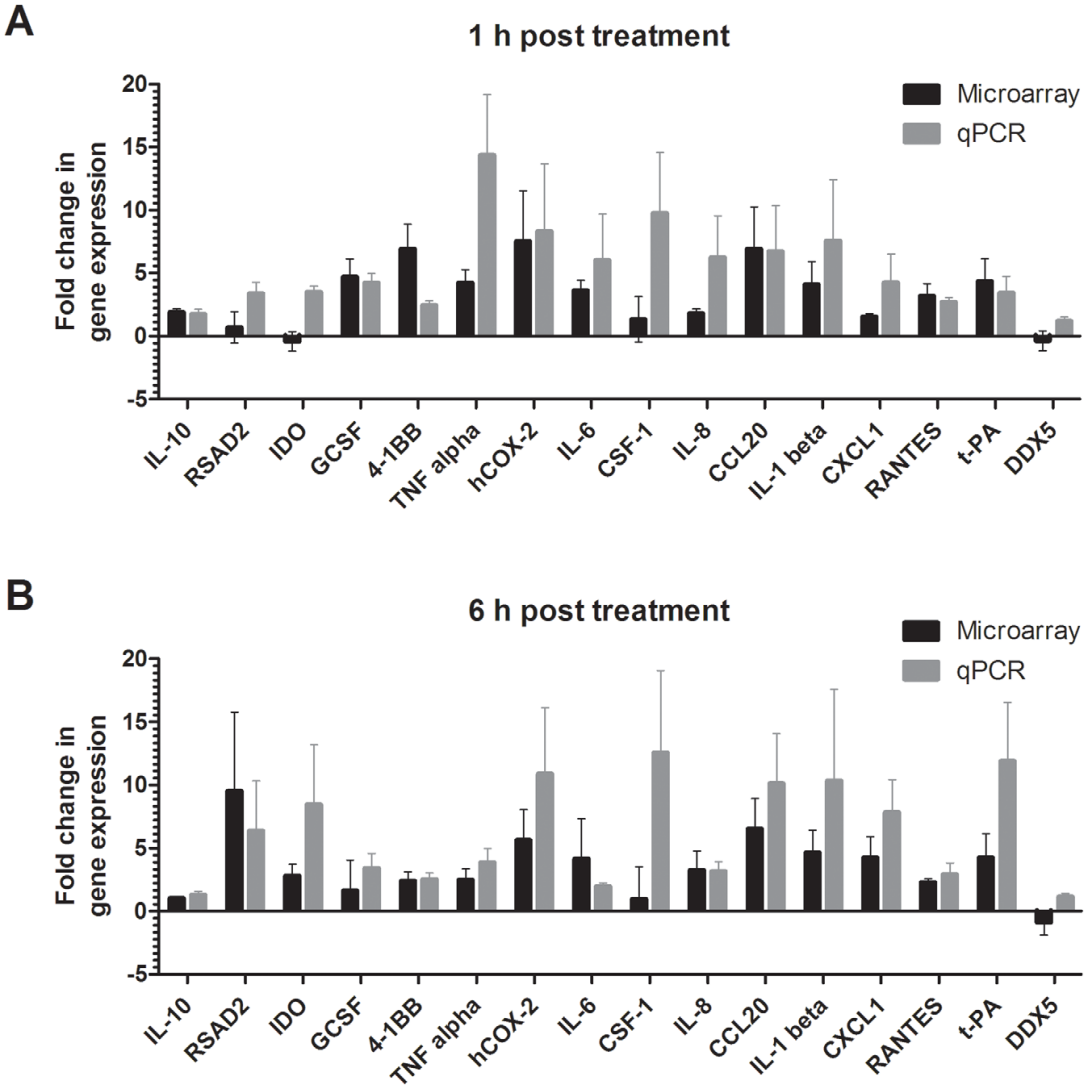
Comparison of DNA microarray and real-time PCR data. DNA microarray results and real-time RT-PCR quantification of changes in cellular expression levels of 16 selected human macrophages genes after 1 h (A) or 6 h (B) post exposure to Ebola virions. Data are expressed as fold-change of cellular gene expression. Data represent the median value for donors 1, 2, and 3.

DNA microarrays are sensitive and specific in identifying regulated transcripts, but the technique commonly leads to underestimation of the degree of fold-change in gene expression. Comparison of the fold-changes in gene expression determined by DNA microarrays to a standardized quantitative real-time RT-PCR demonstrated that the underestimation error was larger when the fold-change differences were higher, corresponding to the finding that the quantitative range of real-time RT-PCR is larger than of DNA microarrays [29]. Consequently, real-time RT-PCR was performed for a subset of 16 selected genes that fulfilled at least one of the selection criteria to verify the results obtained by DNA microarray analysis. Some of the selected genes served as controls and are known to be influenced by ZEBOV infection, however, not necessarily at these early time points (TNF-α, IL-1β, IL-6, IL-8, CXCL1, RANTES, IL-10). Some genes were selected according to their potential relevance in infection due to their known functions and roles in modulation of immune response, viral infections or signaling pathways (hCOX-2, CCL20, 4-1BB, CSF-1, G-CSF, INDO, RSAD2, t-PA, DDX5).Verification was achieved when the direction of gene expression levels was identical and when the expression level changes determined by real-time PCR were equal or stronger than those determined by DNA microarray analysis. Figure 4 illustrates the comparison of the results of microarray analysis and real-time PCR quantifications over both time points. Real-time PCR confirmed the direction of changes in gene expression levels determined by microarray analysis and verified that most changes in cellular gene expression already occurred at the 1 h time point. In particular, upregulation of genes known to play an important role in Ebola hemorrhagic fever (CXCL-1, IL-1β, IL-6, IL-8, and TNF-α [6], [11]) was verified, although some variation was seen among donors.

### Response triggering of macrophages upon ZEBOV binding/entry

**Figure 5:**
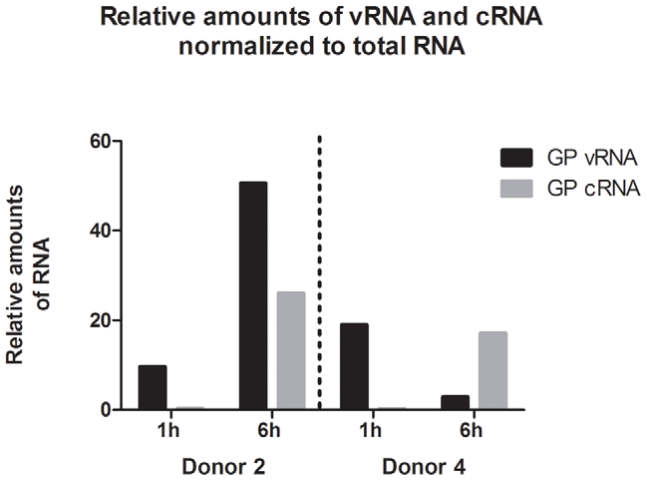
Strand-specific detection ZEBOV RNA. Strand-specific detection of the negative-stranded genomic ZEBOV GP RNA and the positive-stranded ZEBOV GP cRNAs by real-time RT-PCR performed on RNA purified from macrophages of donors D2 and D4 at 1 h and 6 h after virion exposure. All values are normalized to total RNA.

The observation that most cellular responses occurred within 1 h after exposure to Ebola virions suggested that virion binding/entry, rather than expression of virus proteins, initiates them. This hypothesis is based on the absence of virus protein expression during the first hour of virion exposure. To investigate this assumption, real-time RT-PCR quantification, specific for either the negative-stranded ZEBOV genome (vRNA) or for the positive-stranded complementary ZEBOV cRNAs, was performed on RNA purified from macrophages at 1 h and 6 h after virion exposure. The obtained data (Figure 5) confirmed that at 1 h cRNA amounts were below that of genomic RNA, whereas after 6 h cRNA was greatly increased.

**Figure 6:**
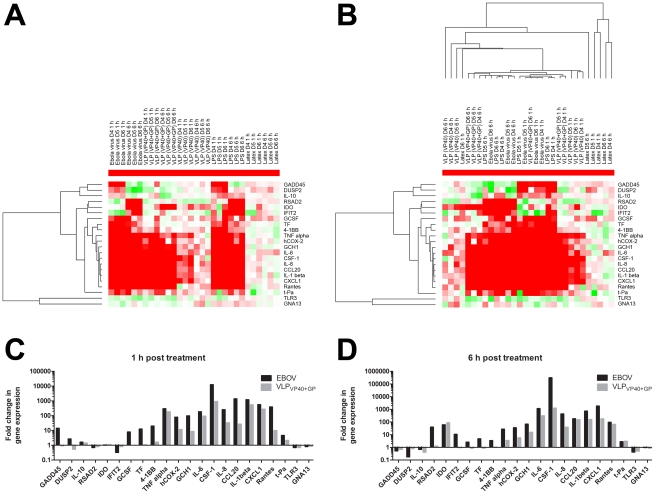
Determination of relative changes in expression levels of 21 cellular genes in primary human macrophages. Depicted are fold-changes at the 1 h and 6 h time points after exposure to Ebola virions compared to mock-exposed cells, cells exposed to purified Ebola virion-like particles (VLPs) containing VP40 and GP_1,2_ (VLP_VP40-GP_) or VLPs containing VP40 only (VLP_VP40_), or cells treated with mock-VLP preparation. As controls, macrophages of each donor were treated in parallel with latex beads or with 10 ng of LPS. (A) Color-coded first dimension hierarchal cluster analysis based on average linkage clustering of the fold-changes in cellular gene expression in cells of donors D4, D5 and D6 at the 1 h and 6 h time points. Ratios were calculated as log_2_ values. Positive values indicate upregulation as compared to mock infection/treatments and are represented by red coloration. Negative values indicate down regulation and are represented by green colors. White indicates no change in gene expression (log_2_ = 0). Each gene has been tested with 5 different stimuli (ZEBOV, VLP_VP40-GP_, VLPs VLP_VP40_, LPS, and neutral latex beads), in 3 donors at 2 time points (1 h and 6 h post infection). This provides a total of 30 different evaluations of changes in expression levels per gene. Blocks of three (1 h or 6 h for 3 donors) or six variables (e.g. VLP) can clearly be seen. (B) Color-coded second-dimensional clustering based on genes with similar changes in expression levels. Fold-changes in cellular gene expression following exposure to Ebola virions or treatment with VLP_VP40-GP_ after 1 h (C) and 6 h (D) incubation of human macrophages from donors D4, D5 and D6. Data represent the median values of the fold-changes in log_2_ of cellular gene expression of donors D4–6.

LPS served as a positive control for the responsiveness of the macrophages used in the virion exposure studies. A comparison of the effects of Ebola virion exposure versus LPS treatment revealed that the majority of genes responded similarly (Supplemental Table 1), further substantiating the hypothesis that the observed responses with Ebola virions were triggered by binding and/or entry. To further investigate which changes in expression levels in macrophages occur due to virion binding/entry, macrophage responses following exposure with infectious virions were directly compared to exposure to Ebola virus-like particles (VLPs) lacking viral genetic material and containing only the ZEBOV matrix protein VP40 and the ZEBOV spike glycoprotein GP_1,2_ (VLP_VP40-GP_) (see also [19]). GP_1,2_ is the only known surface-exposed structural component of Ebola virions and contains the cell-surface receptor-binding domain [30], [31]. We therefore hypothesized that if virion binding is responsible for the observed macrophage responses, then these effects would be mediated by GP_1,2_. To evaluate the role of GP_1,2_, VLPs consisting of VP40, but lacking GP_1,2_ (VLP_VP40_), were tested in parallel with VLP_VP40-GP_. VLP_VP40_ particles were shown previously to be morphologically similar to the GP_1,2_-containing VLPs [32], [33]. LPS was again used as a control for macrophage responsiveness and also to directly compare the LPS-induced responses to those of Ebola virion and VLP exposures. Additionally, cells were exposed to latex particles, which were used in approximate equal quantity to VLPs. Ebola VLP preparations were tested by negative-stain electron microscopy for authentic appearance and presence (VLP_VP40-GP_) or absence (VLP_VP40_) of GP_1,2_ (data not shown). Particles resembled those previously described [32], [33]. In addition, Ebola virion, VLP, and latex preparations were tested for endotoxin-contamination before incubation with macrophages from donors D4, D5, and D6. The endotoxin concentrations of all samples used in this study were not above those of the tissue culture media (<0.5 U/ml). To determine whether the quantitative differences in gene expression levels between cells of donors D1–D3 observed in the first experiment were due to the MOI used, a higher MOI of Ebola virions (≈100) was used for this experiment. Cells were exposed to VLPs and latex particles at a concentration of 100 particles per cell. LPS was used at the same concentration as in the first experiment (10 ng/ml), which allowed for a direct comparison of the responsiveness of macrophages used in both experiments. The results revealed that while a higher MOI did increase the overall intensity of responses compared to the first experiments (compare to Figure 4), it did not eliminate or noticeably reduce the degree of variation of gene expression levels between donors (Figure 6C & 6D). This suggests that genetic variation among identical cell types of different donors may have an influence on the effect of ZEBOV infection and therefore may influence chances of survival.

Since all stimuli were given in parallel to macrophages from each donor, this experiment allowed for a direct qualitative comparison of the cellular responses to the different stimuli. Cellular gene expression levels were determined by real-time RT-PCR specific for 21 genes that were selected from the list of genes established using DNA microarray analysis in the first experiment. These 21 genes included the majority of genes previously analyzed by real time PCR (Figure 4), as well as additional genes that were suspected or known to play a role in ZEBOV infection *in vivo*.

### a) Main clusters

The results of the real-time PCR quantification were analyzed by two-dimensional cluster analysis. The first clustering was performed to group genes according to similar behavior in expression changes (upregulation or downregulation) following a stimulus (Figure 6A). This first cluster was then used for clustering in a second dimension to group experimental stimuli according to similar effects they had on changes in gene expression (Figure 6A, right cluster). Analysis revealed genes that were upregulated at the 1 h and then downregulated at the 6 h time point (GADD45, DUSP2, IL-10), genes that were first downregulated and then upregulated (IDO, ISG 45K), a large group of genes induced at both time points, and finally genes that were downregulated at both time points (TLR3, GNA13). The second clustering ordered the experimental groups according to similar responses in gene expression (Figure 6B) and revealed the following three major clusters: 1) 1 h exposure to Ebola virions, LPS, and VLP_VP40-GP_, 2) 6 h exposure to Ebola virions, LPS, and VLP_VP40-GP_, and 3) 1 h and 6 h exposure to VLP_VP40_ and latex particles. This result suggests that Ebola virions, LPS, and VLP_VP40-GP_ caused strikingly similar responses in comparison to the responses to VLP_VP40_ and latex particles. This further supports the notion that binding/entry of virions mediated by GP_1,2_ plays a significant role in the host response to infection. Thirteen of the 21 genes tested responded to Ebola virions, VLP_VP40-GP_, and LPS at both time points. TNF-α, IL-1β, IL-6 and IL-8 were also found in a separate study on the effects of VLPs on macrophages [19] and CXCL1 represents a gene known to be affected by ZEBOV infection. However, COX-2, GCH1, GM-CSF, MIP-3α, t-Pa, GADD45A, and IDO (6 h) are genes identified to play a role in Ebola hemorrhagic fever for the first time.

### b) Subclusters

Within the first two clusters, various subclusters were identified. One revealed that out of the 13 genes that were upregulated at both time points, 9 responded, albeit weakly, to VLP_VP40_ at the 1 h time point. This supports the notion that the matrix protein VP40 might also be involved in cellular interactions upon binding/entry, which may participate in triggering part of the detected responses in macrophages, mainly involved in proinflammatory signaling. Exposure to latex particles did not induce a response, indicating that the signals mentioned above were not due to non-specific cellular uptake of particles. Another subcluster differentiated macrophages treated with VLP_VP40-GP_ versus Ebola virions or LPS and included IFN-inducible genes such as RSAD2 and IFIT2, as well as G-CSF, TF, GADD45, DUSP2, IL-10, IDO, and CD 137lig. Another sub-cluster revealed 3 genes, DUDP2, IL-10, and TLR3, which were downregulated at the 6 h time point, and one gene, GNA13, which was downregulated at both time points by Ebola virions or VLP_VP40-GP_ but not by LPS.

### c) Mixing clusters

At the 1 h time point, cells responded to Ebola virions and LPS by upregulating GADD45A, DUSP2, and IL-10, whereas these genes were downregulated at the 6 h time point. Both stimuli (virions and LPS) resulted in upregulation at both time points of TF and 4-1BB. The IFN-inducible genes RSAD2 and IFIT2, G-CSF (CSF-3), and GNA13 responded at the 1 h time point to both Ebola virions and VLP_VP40-GP_ and at the 6 h time point to both Ebola virions and LPS. One possible explanation for this delayed response might be differences in the kinetics of the responses between the LPS and virions or VLP_VP40-GP_ stimuli. Of the genes that responded to only two of the presented stimuli, more genes reacted similarly to virions and LPS than to virions and VLP. A direct comparison between responses to virion and VLP_VP40-GP_ exposure is depicted in Figures 6C and D for the 1 h and 6 h time points, respectively. Overall, the cluster analysis and the graphs depicting the real-time RT-PCR quantifications indicate that there was, in general, a significant correlation between the effects of virions and effects of VLP_VP40-GP_ with correlation coefficients of R^2^ of 0.746 and 0.9177 at the 1 h and 6 h time points, respectively. In fact, the difference consists of the expression patterns of G-CSF, 4-1BB, TF, RSAD2 (at 6 h only), and DUSP2, which are the genes identified in the sub-clusters. The real-time RT-PCR also revealed that the changes on gene expression of IL-10, GNA13 and TLR3 are not significantly changed at these early time points even at 100 MOI infection.

## Discussion

The current study represents the first broad analysis of initial transcriptional responses to Ebola virions of human macrophages, the primary target cells of ZEBOV [12], [13], [14], [15]. To assure accurate identification of cellular genes that responded significantly to virion exposure, DNA microarray data were scrutinized and stringently filtered by two different sets of selection criteria and statistical evaluations. In addition, the expression levels of selected genes were experimentally verified by real-time PCR analysis. Using these methods for statistical verification and assay evaluation, our study permitted the identification of a large number of genes not previously implicated in early cellular responses to ZEBOV infection. The detected changes of gene expression levels occurred within the first hours after primary human macrophages were exposed to Ebola virions *in vitro*. In addition, we identified genes whose expression is known to be upregulated during acute Ebola hemorrhagic fever. This not only validated the experimental approach and data screening criteria, it also demonstrated that elevated levels of gene products, such as cytokines, are already induced in primary target cells within the first hours of virion binding and entry. Lending further credence to these assertions, pathway ORA of the significantly differentially regulated genes identified from our gene expression studies demonstrated a strong upregulation of specific host signaling pathways related to cytokine/chemokine signaling during the immediate response to Ebola infection. Indeed, the upregulation of signaling pathways related to cytokine/chemokine signaling events suggests that specific innate immune responses are mounted during the acute phase of Ebola viral insult. Through multiple pathway ORA analyses we identified broad cellular functional networks that are modulated during the early course of Ebola infection and, importantly, have correlated this with specific cell signaling pathways. The identities of the individual signaling pathways modulated by Ebola infection will provide critical information regarding disease pathogenesis and as well information for the development of novel antiviral therapeutics.

Some of the genes that had already been implicated in the pathogenesis of Ebola hemorrhagic fever such as IL-6 and TNF-α [6], [11] were induced after 1 h, but returned towards basal levels of expression after 6 h. Indeed, whereas LPS stimulation resulted in the activation of a large subset of TNF-α related signaling pathways at the 6 h time point there were no common pathways identified in the Ebola-infected pathway ORA. Similar results were obtained in another study, during which primary human macrophages, exposed to Ebola VLP_VP40-GP_ for 24 h, were characterized by IL-1β, IL-6, IL-8, RANTES, and TNF-α expression that also peaked at 1 h or 6 h before receding to base levels [19]. These results contradict observations *in vivo*, which demonstrated upregulation of cytokines for extended periods of time [6], [7], [8], [9], [10], [11]. It is plausible that the early cytokine peak observed following high MOI infections *in vitro* is only achieved at a later time during *in vivo* infection. Another possible explanation for this difference is the duration of stimuli and thereby the duration of responses. For instance, *in vivo*, progeny virions are continuously produced by ZEBOV-infected cells and will therefore bind to and enter additional target cells, resulting in continuous stimuli that may maintain cytokine production and facilitate prolonged activation. Therefore, our *in vitro* approach most likely yielded results that are indicative of what continuously occurs *in vivo*. That increased cellular gene expression levels result in increased protein concentrations within 6 h was previously demonstrated in a study that used human macrophages exposed to VLP_VP40-GP_ and ELISA measuring protein levels of IL-1β, IL-6, IL-8, TNF-α [19].

The identified cellular genes whose expression levels were altered after exposure to Ebola virions belonged to different functional categories (Table S1). These genes include inflammatory cytokines, molecules that regulate blood coagulation (such as t-Pa, MMP-1, and serpine 1 and 2) genes involved in stress-response, DNA repair, cell cycle arrest, and cell adhesion. In support of these functional categories, our pathway ORA also resulted in similar functional categorization of the differentially regulated signaling pathways following Ebola virus infection. The detection of a group of genes that responded to binding/entry of Ebola virions, VLP_VP40-GP_, as well as to LPS raises the question whether LPS and Ebola virions share receptors on the macrophage surface. Whereas receptors for Ebola virions on target cells remain elusive, it is known that LPS induces its effects by binding to TLR4 and MD2 and CD14 co-receptors. Recent studies demonstrated that Ebola VLP_VP40-GP_, but not VLP_VP40_, induced cytokine and SOCS1 expression in a TLR4/MD2 dependent manner both in a human monocytic cell line (THP-1 cells) and in 293T cells expressing a functional TLR4/MD2 receptor [34]. The innate immune defense is achieved by activating NF-κB and type I IFN responses. It is already known that ZEBOV suppresses the host cell antiviral response by inhibiting interferon signaling via its VP35 and VP24 proteins [35], [36], [37], [38], [39], [40], [41], and RNA silencing via VP35 [42]. Our studies revealed only limited IFN signaling in Ebola virion-exposed macrophages compared to those exposed to LPS, indicating that IFN signaling is inhibited early upon infection.

The host response to virulent pathogens is likely to fall into two categories [21]. First, there are common responses to unrelated pathogens, such as the type 1 IFN innate immune response that renders uninfected neighboring cells resistant to virus infection. Second, there are responses specific for individual pathogens. On the other hand, pathogens have evolved to counter these responses to ensure their own survival and transmission. Examples are how viruses of disparate families overcome the antiviral action of apolipoprotein B mRNA editing enzyme, catalytic polypeptide-like 3G (APOBEC3G) [43], tripartite motif containing 5 (TRIM5α) [44], bone marrow stromal cell antigen 2 (BST-2)/tetherin [45], [46], [47], or interferon induced transmembrane proteins (IFITM) [46], [48], [49]. It is important to remember that this study partially characterizes the response of humans to ZEBOV infection and that humans are not natural hosts of this virus. Therefore, host responses and virus counteractions are not in a state of equilibrium. It is therefore a fundamentally interesting question whether infected humans succumb to Ebola hemorrhagic fever because of direct effects exerted by the virus on the body or because of overbearing immune responses by the individual. Recently, various frugivorous bats have been implicated as potential filovirus reservoirs that seemingly remain unaffected by infection [50], [51]. It is therefore tempting to repeat our studies with cells from these animals to see whether their responses to Ebola virion exposure are fundamentally different.

Taking together, our data indicate that the immediate responses of early cellular ZEBOV targets in the human organism derive from virion binding/entry mediated by the ZEBOV spike glycoprotein and do not require virus gene expression. The fact that many surveyed genes responded similarly to VLP_VP40-GP_, but not to VLP_VP40_, treatment clearly supports this notion. However, the fact that some genes, such as those encoding TF or skin collagenase, were triggered only by Ebola virions but not VLP_VP40-GP_ or LPS indicate that these genes must be influenced by factors other than virus binding or entry, such as other components packaged within Ebola virions in addition to GP_1,2_ and VP40. In this regard, it is important to remember that infectious Ebola virions not only consist of seven structural proteins (NP, VP35, VP40, GP_1,2_, VP30, VP24, and L) but also contain cellular transmembrane proteins that are usurped by budding virions [52].

## Supporting Information

Figure S1 **MA plot depicting the overall distribution of gene expression changes as a function of average signal intensity for human macrophages obtained from three different donors.** Each data set compares gene expression levels and changes in primary macrophages exposed to Ebola virions compared to mock-exposure at 1 h (A) and 6 h (B).
(TIF)

Table S1 **Summary of genes identified using threshold and trend analysis.** 88 genes were identified from both threshold and trend analysis. Genes are grouped according to their known functions, determined by GeneOntology descriptors [53]. Many of these genes express proteins involved in apoptosis, inflammatory and acute immune responses, blood coagulation, and tissue remodeling.
(DOCX)
