# Supplementary figures and images for: Ebola Virion Attachment and Entry into Human Macrophages Profoundly Effects Early Cellular Gene Expression
Source: PLoS Negl Trop Dis. 2011 Oct 18;5(10):e1359. doi: 10.1371/journal.pntd.0001359 (PMC3196478; doi:10.1371/journal.pntd.0001359)

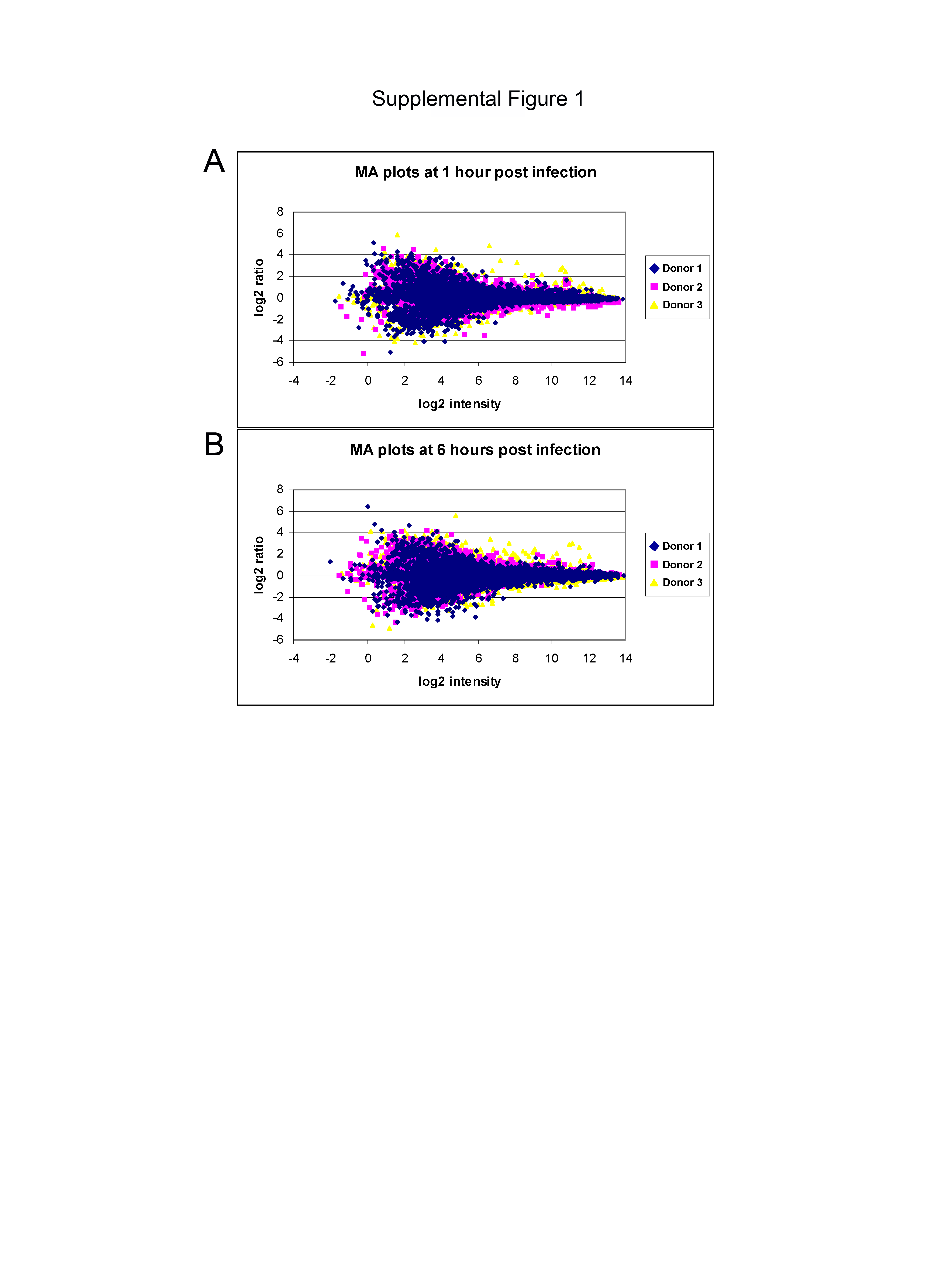

Supplement: Figure S1 — MA plot depicting the overall distribution of gene expression changes as a function of average signal intensity for human macrophages obtained from three different donors. Each data set compares gene expression levels and changes in primary macrophages exposed to Ebola virions compared to mock-exposure at 1 h (A) and 6 h (B). (TIF) [file pntd.0001359.s001.tif]
