# Supplementary material for: Ebola Virion Attachment and Entry into Human Macrophages Profoundly Effects Early Cellular Gene Expression
Source: PLoS Negl Trop Dis. 2011 Oct 18;5(10):e1359. doi: 10.1371/journal.pntd.0001359 (PMC3196478; doi:10.1371/journal.pntd.0001359)
Supplement: Table S1 — Summary of genes identified using threshold and trend analysis. 88 genes were identified from both threshold and trend analysis. Genes are grouped according to their known functions, determined by GeneOntology descriptors [53]. Many of these genes express proteins involved in apoptosis, inflammatory and acute immune responses, blood coagulation, and tissue remodeling. (DOCX) [file pntd.0001359.s002.docx]

**Table S1. Summary of genes identified using threshold and trend analysis.**

| **Gene Ontology Biological Process** | **Accession number** | **Gene title** | **Gene symbol** | **Average change fold at 1h microarray** | **Average change fold at 6h microarray** | **Selection criteria 1 (threshold)** | **Selection criteria 2 (trend)** | **LPS response** | **PCR tested** |
| --- | --- | --- | --- | --- | --- | --- | --- | --- | --- |
| --- | L20861 | wingless-type MMTV integration site family, member 5A | WNT5A | 2.1 | 2.1 | X | X |  |  |
| --- | D21205 | tripartite motif-containing 25 | TRIM25 | 2.1 | 1.8 | X |  |  |  |
| --- | M36821 | chemokine (C-X-C motif) ligand 3 | CXCL3 | 1.8 | 2.4 |  | X | X |  |
| --- | AF001434 | EH-domain containing 1 | EHD1 | 1.6 | 1.4 |  | X | X |  |
| --- | D31770 | activin A receptor, type II | ACVR2 | 1.4 | 0.9 |  | X | X (DOWN) |  |
| --- | AB000115 | chromosome 1 open reading frame 29 | C1orf29 | 1.3 | 1.5 | X |  | X |  |
| --- | AF026941 | vipirin, cytomegalovirus-induced gene | cig5 | 1.2 | 2.8 | X |  | X | X |
| --- | AB029014 | RAB6 interacting protein 1 | RAB6IP1 | 1.1 | 1.4 |  | X | X |  |
| --- | HG1996-HT2044 | --- | --- | 0.7 | 0.3 | X |  |  |  |
| --- | AF000561 | zinc finger and BTB domain containing 7 | ZBTB7 | 0.6 | 0.4 | X |  |  |  |
|  |  |  |  |  |  |  |  |  |  |
| **apoptosis / inflammatory response** | X02910 | tumor necrosis factor (TNF superfamily, member 2) | TNF | 4.1 | 1.9 | X | X | X | X |
| apoptosis / inflammatory response | M16441 | tumor necrosis factor (TNF superfamily, member 2) | TNF, LTA | 3.0 | 1.3 | X | X |  |  |
| apoptosis / cell cycle / antimicrobial humoral response | J05008 | interleukin 1, beta | IL1B | 2.9 | 3.6 | X | X | X | X |
| apoptosis / inflammatory response | AF054176 | cold autoinflammatory syndrome 1 | CIAS1 | 2.6 | 1.0 | X | X |  |  |
| apoptosis / immune response | U03398 | tumor necrosis factor (ligand) superfamily, member 9 | TNFSF9 | 2.5 | 1.0 | X | X |  | X |
| apoptosis | M59465 | tumor necrosis factor, alpha-induced protein 3 | TNFAIP3 | 2.0 | 1.3 | X | X | X |  |
| apoptosis / morphogenesis | S81914 | immediate early response 3 | IER3 | 2.0 | 2.5 | X | X | X |  |
| anti-apoptosis / immune response / neg. regulation of nitric oxide biosynthesis | U16720 | Human interleukin 10 | IL10 | 1.8 | 1.1 |  | X | X | X |
| apoptosis / anti-apoptosis | Y00630 | serine (or cysteine) proteinase inhibitor, clade B (ovalbumin), member 2 | SERPINB2 | 1.6 | 2.7 | X |  | X |  |
| apoptosis | U45878 | baculoviral IAP repeat-containing 3 | BIRC3 | 1.6 | 1.6 |  | X | X |  |
| apoptosis / protein complex assembly | U19261 | TNF receptor-associated factor 1 | TRAF1 | 1.5 | 1.7 |  | X | X |  |
| apoptosis | AF001294 | pleckstrin homology-like domain, family A, member 2 | PHLDA2 | 1.4 | 1.5 |  | X | X |  |
| apoptosis / anti-apoptosis | AF099935 | tumor necrosis factor, alpha-induced protein 8 | TNFAIP8 | 1.4 | 1.1 |  | X | X |  |
|  |  |  |  |  |  |  |  |  |  |
| **blood coagulation / proteolysis and peptidolysis** | M15518 | plasminogen activator, tissue-type | PLAT | 1.7 | 2.0 | X |  | X | X |
| blood / angiogenesis | M92357 | tumor necrosis factor, alpha-induced protein 2 | TNFAIP2 | 1.6 | 1.5 |  | X | X |  |
| blood coagulation | M14083 | serine (or cysteine) proteinase inhibitor, clade E (nexin) member 1 | SERPINE1 | 1.4 | 0.9 |  | X |  |  |
| collagen catabolism | M13509 | matrix metalloproteinase 1 (interstitial collagenase) | MMP1 | 1.3 | 2.2 | X | X | X | (X) |
|  |  |  |  |  |  |  |  |  |  |
| **cell adhesion / inflammatory response** | M24736 | selectin E (endothelial adhesion molecule 1) | SELE | 2.6 | 1.0 | X | X |  |  |
| cell adhesion / inflammatory response | M31165 | tumor necrosis factor, alpha-induced protein 6 | TNFAIP6 | 2.4 | 2.1 | X | X | X |  |
| cell adhesion | M24283 | intercellular adhesion molecule 1 (CD54), human rhinovirus receptor | ICAM1 | 1.6 | 1.4 |  | X | X |  |
| cell adhesion | X78565 | tenascin C (hexabrachion) | TNC | 1.1 | 1.8 | X |  | X |  |
| cell adhesion | L05424 | CD44 antigen (homing function and Indian blood group system) | CD44 | 1.1 | 1.6 |  | X | X |  |
| cell adhesion / immune response | X60992 | CD6 antigen | CD6 | 1.0 | 0.3 | X |  |  |  |
|  |  |  |  |  |  |  |  |  |  |
| **cell cycle arrest / DNA repair** | M60974 | growth arrest and DNA-damage-inducible, alpha | GADD45A | 2.1 | 0.9 |  | X |  | X |
| cell proliferation | AB013924 | lysosomal-associated membrane protein 3 | LAMP3 | 1.9 | 2.1 | X | X | X |  |
| cell cycle / angiogenesis / cell-cell signaling | D30783 | epiregulin | EREG | 1.7 | 2.6 |  | X |  |  |
| cell cycle checkpoint | AF058696 | Nijmegen breakage syndrome 1 (nibrin) | NBS1 | 1.1 | 1.3 |  | X | X |  |
| cell proliferation / antimicrobial humoral response | U33017 | signaling lymphocytic activation molecule family member 1 | SLAMF1 | 0.9 | 2.0 | X | X | X |  |
| cell proliferation / development | AF062739 | frequently rearranged in advanced T-cell lymphomas 2 | FRAT2 | 0.9 | 0.5 | X |  |  |  |
| cell cycle / chemotaxis | M54995 | pro-platelet basic protein (chemokine (C-X-C motif) ligand 7) | PPBP | 0.6 | 1.2 | X |  |  |  |
|  |  |  |  |  |  |  |  |  |  |
| **chemotaxis / inflammatory response** | U6419 | macrophage inflammatory protein, liver & activation-regulated chemokine | CCL20 | 4.8 | 4.8 | X | X | X | X |
| cell motility / inflammatory response | U04636 | prostaglandin-endoperoxide synthase 2 (G/H synthase and cyclooxygenase) | PTGS2 | 4.3 | 3.8 | X | X | X | X |
| calcium ion homeostasis / cell motility / cellular defense response / response to oxidative stress | M21121 | chemokine (C-C motif) ligand 5 | CCL5 | 2.3 | 2.1 | X | X | X | X |
|  | U81234 | chemokine (C-X-C motif) ligand 6 (granulocyte chemotactic protein 2) | CXCL6 | 2.1 | 5.5 | X |  |  |  |
|  | M36820 | chemokine (C-X-C motif) ligand 2 | CXCL2 | 2.0 | 2.7 | X | X | X |  |
| angiogenesis / cell motility / calcium-mediated signaling / neutrophil activation / regulation of retroviral replication | M28130 | Human interleukin 8 | IL8 | 1.6 | 2.7 | X | X | X | X |
|  | X54489 | chemokine (C-X-C motif) ligand 2, melanoma growth stimulatory activity | CXCL2 | 1.5 | 3.2 | X | X | X | X |
| cell motility / cell growth and/or maintenance / viral genome replication | J04130 | chemokine (C-C motif) ligand 4 | CCL4 | 1.4 | 2.0 |  | X | X |  |
| cytosolic calcium ion concentration elevation / antimicrobial humoral response | L31584 | chemokine (C-C motif) receptor 7 | CCR7 | 1.4 | 1.6 |  | X | X |  |
| cell motility / calcium ion homeostasis / antimicrobial humoral response | D90144 | chemokine (C-C motif) ligand 3 | CCL3 | 1.3 | 1.9 |  | X | X |  |
| calcium ion homeostasis | AF088219 | chemokine (C-C motif) ligand 23 | CCL23 | 1.1 | 1.6 |  | X |  |  |
| calcium ion transport / | Y16645 | chemokine (C-C motif) ligand 8 | CCL8 | 1.1 | 2.4 | X |  | X |  |
| cell motility | L22075 | guanine nucleotide binding protein (G protein), alpha 13 | GNA13 | 0.7 | 1.1 |  | X | X (UP) | X |
|  |  |  |  |  |  |  |  |  |  |
| **immune response / acute-phase response** | X04430 | interleukin 6 (interferon, beta 2) | IL6 | 5.4 | 2.0 | X | X | X | X |
|  | M31166 | pentaxin-related gene, rapidly induced by IL-1 beta | PTX3 | 3.3 | 2.0 | X | X | X |  |
| defense response, cellular | M13207 | colony stimulating factor 2 (granulocyte-macrophage) | CSF2 | 3.0 | 1.3 | X |  |  | X |
| defense response | X03656 | colony stimulating factor 3 (granulocyte) | CSF3 | 2.2 | 1.2 | X |  |  | X |
| defense response, cellular | M13207 | colony stimulating factor 2 (granulocyte-macrophage) | CSF2 | 1.8 | 2.0 | X |  |  | X |
|  | M14660 | interferon-induced protein with tetratricopeptide repeats 2 | IFIT2 | 1.6 | 0.9 | X |  | X | X |
|  | M55542 | guanylate binding protein 1, interferon-inducible, 67kDa | GBP1 | 1.1 | 1.6 |  | X | X |  |
|  | AF026939 | interferon-induced protein with tetratricopeptide repeats 4 | IFIT4 | 1.1 | 1.7 | X |  | X |  |
|  | AJ225089 | 2'-5'-oligoadenylate synthetase-like | OASL | 1.1 | 1.6 | X |  | X |  |
|  | M24594 | interferon-induced protein with tetratricopeptide repeats 1 | IFIT1 | 1.0 | 2.2 | X |  | X |  |
|  | AF004231 | leukocyte immunoglobulin-like receptor, subfamily B , member 2 | LILRB2 | 0.6 | 1.6 |  | X | X |  |
| defense response to bacteria/ inflammatory response / detection of virus | U88879 | toll-like receptor 3 | TLR3 | 0.3 | 1.2 | X |  | X (UP) | X |
|  |  |  |  |  |  |  |  |  |  |
| **metabolism / mRNA catabolism** | M92843 | zinc finger protein 36, C3H type, homolog (mouse) | ZFP36 | 1.9 | 1.2 |  | X | X |  |
| cAMP biosynthesis / progesterone biosynthesis / response to wounding | D14874 | adrenomedullin | ADM | 1.8 | 1.3 |  | X | X |  |
| nucleotide metabolism / purine ribonucleoside monophosphate biosynthesis / antimicrobial humoral response | X02994 | adenosine deaminase | ADA | 1.3 | 1.4 |  | X | X |  |
| phenylalanine catabolism / nitric oxide biosynthesis | U19523 | GTP cyclohydrolase 1 (dopa-responsive dystonia) | GCH1 | 1.3 | 1.7 |  | X | X |  |
| tryptophan catabolism / immune response | M34455 | indoleamine-pyrrole 2,3 dioxygenase | INDO | 1.2 | 2.4 | X | X | X | X |
| fructose 2,6-bisphosphate metabolism | D49817 | 6-phosphofructo-2-kinase/fructose-2,6-biphosphatase 3 | PFKFB3 | 1.1 | 1.4 | X |  |  |  |
| oxidative stress / superoxide metabolism | X07834 | superoxide dismutase 2, mitochondrial | SOD2 | 1.0 | 1.9 | X |  | X |  |
| protein biosynthesis | AI768188 | mitochondrial ribosomal protein S6 | MRPS6 | 1.0 | 0.7 |  | X | X |  |
|  |  |  |  |  |  |  |  |  |  |
| **signal transduction / protein amino acid dephosphorylation / response to oxidative stress** | X68277 | dual specificity phosphatase 1 | DUSP1 | 2.5 | 1.3 | X | X | X |  |
|  | L11329 | dual specificity phosphatase 2, phoshatase of activated cells 1 | DUSP2 | 2.0 | 0.9 |  | X | X (DOWN) | X |
| cell proliferation | AF035279 | interleukin 15 receptor, alpha | IL15RA | 1.7 | 2.2 | X | X | X |  |
| protein amino acid dephosphorylation | U15932 | dual specificity phosphatase 5 | DUSP5 | 1.6 | 1.4 |  | X | X |  |
|  | L20971 | phosphodiesterase 4B, cAMP-specific | PDE4B | 1.6 | 2.4 | X | X | X |  |
| transcription, DNA-dependent | AF087036 | musculin (activated B-cell factor-1) | MSC | 1.6 | 1.3 |  | X | X |  |
| transcription, DNA-dependent / immune response | X64318 | nuclear factor, interleukin 3 regulated | NFIL3 | 1.4 | 0.9 |  | X |  |  |
| GPCR | AF014958 | chemokine (C-C motif) receptor-like 2 | CCRL2 | 1.3 | 1.0 |  | X | X |  |
| transcription, DNA-dependent | AL021977 | v-maf musculoaponeurotic fibrosarcoma oncogene homolog F (avian) | MAFF | 1.3 | 1.0 |  | X | X (DOWN) |  |
| cell proliferation | U31628 | interleukin 15 receptor, alpha | IL15RA | 1.7 | 2.2 | X | X | X |  |
| cell proliferation / pyridine nucleotide biosynthesis | U02020 | pre-B-cell colony enhancing factor 1 | PBEF1 | 1.1 | 2.2 |  | X | X |  |
| transcription, DNA-dependent | S68271 | cAMP responsive element modulator | CREM | 0.8 | 1.0 |  | X |  | X |
| transcription, DNA-dependent | AI693307 | MAX protein | MAX | 0.4 | 0.9 | X |  |  |  |
